# Computer-assisted cognitive behavior therapy for obsessive-compulsive disorder: a randomized trial on the impact of lay vs. professional coaching

**DOI:** 10.1186/s12991-015-0048-0

**Published:** 2015-02-22

**Authors:** Kenneth A Kobak, Revere Greist, David M Jacobi, Hollie Levy-Mack, John H Greist

**Affiliations:** Center for Telepsychology, 7601 Ganser Way, Madison, WI 53719 USA; Waypoint Health Innovations, 137 E. Wilson Street, Suite 812, Madison, WI 53703 USA; Rogers Memorial Hospital, 34700 Valley Rd, Oconomowoc, WI 53066 USA; 255 S. 17th Street, Suite 1111, Philadelphia, PA 19103 USA; Healthcare Technology Systems, 6515 Grand Teton Plaza, Suite 100, Madison, WI 53719 USA

**Keywords:** Computerized self-instruction program, Cognitive behavior therapy, Obsessive compulsive disorder

## Abstract

**Background:**

The purpose of the study was to examine the impact of computerized cognitive behavior therapy (CBT) self-help treatment for obsessive-compulsive disorder (OCD) (BT Steps) both alone and when supported by coaching from either a lay non-therapist coach or an experienced CBT therapist.

**Methods:**

Eighty-seven subjects with clinically significant OCD were recruited through newspaper ads and randomly assigned to receive 12 weeks of treatment with either BT Steps alone (*n* = 28), BT Steps with non-therapist coaching (*n* = 28), or BT Steps with CBT therapist coaching (*n* = 31). Subjects worked on BT Steps at their own pace. Subjects receiving BT Steps alone received a welcome call from the project manager. Subjects randomized to either of the coaching arms received regularly scheduled weekly phone calls for coaching, encouragement, and support. No formal therapy was provided by the coaches; thus, both lay and CBT coaches completed the same tasks.

**Results:**

All three treatment arms showed a significant reduction in Yale-Brown Obsessive Compulsive Scale (YBOCS) scores, with mean (SD) changes of 6.5 (5.7), 7.1 (6.1), and 6.5 (6.1) for the no coaching, lay coaching, and therapist coaching arms, respectively (all *p*’s < .001). These represent effect sizes of 1.16, 1.41, and 1.12, respectively. No significant differences were found between treatment arms on YBOCS change scores, *F*(2) = 0.10, *p* = .904, or number of exposures sessions done (*F*(2) = 0.033, *p* = .967). When asked which method of therapy (computer vs. clinician) they preferred, 48% said computer, 33% said face-to-face therapy, and 19% had no preference.

**Conclusions:**

Results support the use of online self-help for the treatment of moderate OCD. The addition of coaching by either a lay coach or a CBT therapist coach did not significantly improve outcomes.

## Introduction

Obsessive-compulsive disorder (OCD), once thought to be a rare condition refractory to treatment, is now known to be surprisingly common. The lifetime prevalence in the US for adults is 2.6% [1] with a 1-year prevalence of 1% [2]. OCD is classified as a severe mental illness by the National Advisory Mental Health Council [3,4] and can be incapacitating, ranking 11th among all medical diseases for disability [5]. Direct and indirect costs of OCD have been estimated to be $8.4 billion [6]. Performing rituals may become a major life activity, severely interfering with one’s job, marriage, or other social relationships [7-9]. Comorbid depression is common, with increased levels of suicidal ideation and attempts [10]. OCD has been reclassified in the *Diagnostic and Statistical Manual of Mental Disorders Fifth Edition* [11] (DSM5) from an anxiety disorder to a new category of disorders, obsessive-compulsive and related disorders [11]. This was based in part on data from studies on symptom phenomenology, treatment response, familialty, genetics, neurocircuitry, and cognitive functioning (see Stein et al. [12] for a review). Brain imaging studies have found a unique pattern of neuronal activation not present in other anxiety disorders (i.e., baseline hyperactivity and hyper-response in the lateral orbitofrontal cortex, anterior cingulate cortex, and caudate). Unique deficits in cognitive functioning have also been found, involving difficulties with cognitive flexibility and response inhibition [13,14]. OCD is one of the first disorders to be reclassified based in part upon biomarker evidence, and thus in line with current trends in the classification of mental disorders as outlined by Research Diagnostic Criteria (RDoC) [15].

Cognitive behavior therapy (CBT) with exposure and ritual prevention (E & RP) is recommended as a first-line treatment for OCD by clinical practice guidelines in both the US and abroad [16-18]. Several decades of research support both its *efficacy* and *effectiveness* [19-23]. It has effect sizes as large as pharmacological interventions [24], with lower relapse rates [25-27]. CBT is considered an empirically supported treatment (EST), i.e., a treatment shown to be efficacious in controlled research with a defined population [28]. There has been a growing emphasis on the need for ESTs from both legal, ethical, and economic perspectives [29-31], and professional and ethical guidelines now require therapists to integrate ESTs into their practice [30,31]. However, with this increased emphasis has come increased demand. As a result, the number of therapists trained in CBT falls far short of the demand [32,33]. The percentage of patients with OCD actually receiving CBT treatment ranges from 5% to 7% [34,35], in spite of the fact that persons with OCD have been found to prefer CBT (either alone or in combination with medication) over treatment with medication alone [36]. Sixty percent of OCD patients receive no treatment at all, and the gap between onset of symptoms and effective treatment averages 17 years [37].

New technologies may provide an opportunity to help solve this problem [38]. A growing body of research has found that computerized self-administered CBT (CCBT) is highly effective, achieving clinical improvements similar to those obtained with therapist-administered CBT [39-42]. A recent meta-analysis of randomized, controlled trials of CCBT for anxiety or depressive disorders found a substantial effect size for CCBT (*g* = 0.77, 95% confidence interval (CI) 0.59–0.95) [41], with gains sustained up to 3 years after treatment [43]. Studies of CCBT for OCD have found it to be an effective treatment [44-48], with effect sizes similar to those found with therapist-administered CBT [44,47,49,50]. Gains were maintained at 3- and 4-month follow-up. CCBT can be offered at a reduced cost, cited by patients in one study as the main barrier to seeking treatment [51]. Patients can work at their own pace on their own schedule, and CCBT provides a treatment option for those who fail to seek treatment due to fears of social stigma [52-54]. A recent survey of OCD patients found that they considered CCBT an acceptable form of treatment, with the most common advantages reported being reduced time (67%), no need for travel (63%), reduced costs (60%), and privacy/anonymity (56%) [55]. Only 10% reported preferring face-to-face treatment. Economic studies of CCBT for OCD have found it to be a cost-effective treatment compared to therapist-administered CBT [56,57].

As with any treatment, adherence is required for the treatment to exert its effect. Our previous work on CCBT for OCD found a clear dose–response relationship: patients who did more CBT homework sessions had greater decreases in symptoms [46]. Studies have found that “computer-assisted” programs (CCBT with limited human contact) fare better than fully “computerized” (no human contact) programs, the former being associated with higher treatment adherence and lower dropout rates [53]. Adding human ‘coaching’ to computer-administered CBT treatments has been found to enhance treatment compliance, patient satisfaction, and outcomes [46,53,58,59]. Human coaching appears to be effective in motivating patients to actually confront their fears using the exposure therapy techniques they learn in the self-help treatment programs and to complete their between session homework assignments. This ‘hybrid’ model of computerized self-help combined with human coaching has been endorsed and implemented by the United Kingdom National Health Service—the first governmental regulatory body to recommend web-based self-help CBT treatment [60]. It utilizes a model with limited human contact with non-therapist coaches. However, the level of human support and whether coaching is done by a therapist or non-therapist have not been empirically examined [53]. Using trained non-therapist ‘coaches’ may be a cost-effective means of improving outcomes. According to ‘stepped care’ models of treatment, matching the appropriate level of intervention, starting with the least restrictive and most effective, enhances treatment outcomes, controls healthcare costs, and helps allocate scarce mental health resources more effectively [61-64]. Recent studies of stepped care in OCD found equivalent treatment outcomes to standard clinical CBT but significantly lower treatment costs [65-67]. While CCBT fits nicely in the stepped care model, understanding the differences in treatment outcome associated with lay vs. therapist CCBT coaches will help inform the stepped care model. This goal of this study was to examine the impact of computerized self-help treatment for OCD alone and in combination with either a lay non-therapist coach or coaching provided by an experienced CBT therapist.

## Method

### Subjects

Eighty-seven subjects with a minimum Yale-Brown Obsessive-Compulsive Scale (YBOCS) [68] score of 16 and a maximum score of 32 were enrolled. This range includes clinically significant OCD but not highest severity. Subjects were recruited through newspaper ads and from referrals from community therapists from January 2012 through December 2013. Subjects were excluded if they had psychotic symptoms, significant comorbid depression (defined as score of 20 or greater on the computer-administered Hamilton Depression Rating Scale [69]), or if they were a serious suicide risk (as measured by a score of 3 or greater on ideation on the Columbia Suicidality Severity Rating Scale (C-SSRS) [70] or any suicidal behavior endorsed on the C-SSRS). Seventy-two percent of subjects had at least one comorbid psychiatric diagnosis as determined by the Structured Clinical Interviews for DSM-IV [71] (SCID) (substance abuse = 6, eating disorders = 2, major depression = 11, dysthymia = 4, depression not otherwise specified = 9, panic disorder = 9, generalized anxiety disorder = 9, posttraumatic stress disorder = 4, social phobia = 10, bipolar disorder = 3, attention-deficit/hyperactivity disorder = 6).

The study was reviewed and approved by the Allendale Institutional Review Board, and all subjects signed informed consent statements approved by the board. Demographic data on the subjects are presented in Table 1. A significantly greater percentage of subjects in the therapist coaching arm had at least a 2-year college degree (*χ*^2^(2) = 6.42, *p* = .04). There were no other significant differences between patients in the treatment arms on any other demographic variables at baseline. Thirty-four subjects (38.6%) were currently in treatment for OCD (medication alone = 13, psychotherapy alone = 4, combination medication and psychoherapy = 16). Average duration of current treatment was 5.4 years. Of the 20 subjects receiving psychotherapy, 14 reported that their therapist uses some CBT techniques as part of their approach. There were no significant differences between groups on the percentage of patients receiving concurrent psychotherapy, (*χ*^2^(2) = 1.41, *p* = .49).

**Table 1:** **Demographic data by treatment arm**

	**College degree** ^**a**^	**Percent female** ^**b**^	**Mean (SD) age** ^**c**^	**Percent Caucasian** ^**d**^	**Mean (SD) baseline YBOCS** ^**e**^	**Currently in OCD treatment** ^**f**^
No coaching (*n* = 28)	64% (*n* = 18)	61% (*n* = 17)	35.43 (14.27)	89% (*n* = 25)	22.82 (3.68)	39% (*n* = 11)
Lay coaching (*n* = 28)	54% (*n* = 15)	61% (*n* = 17)	40.93 (14.09)	89% (*n* = 25)	22.71 (3.97)	36% (*n* = 10)
Therapist coaching (*n* = 31)	83% (*n* = 26)	68% (*n* = 21)	38.65 (13.44)	87% (*n* = 27)	21.81 (4.05)	42% (*n* = 13)
All subjects (*N* = 87)	68% (*n* = 59)	63% (*n* = 55)	38.34 (13.93)	89% (*n* = 77)	22.43 (3.89)	39% (*n* = 34)

^a^At least a 2-year college degree; *χ* ^2^(2) = 6.424, *p* = .04.

^b^ *χ* ^2^(2) = 0.424, *p* = .809.

^c^ *F*(2) = 0.033, *p* = .967.

^d^ *χ* ^2^(2) = 0.94, *p* = .954.

^e^ *F*(2) = 0.101, *p* = .904.

^f^ *χ* ^2^(2) = 0.240, *p* = .887.

### Procedure

Subjects were randomly assigned to receive 12 weeks of treatment in one of three treatment arms: BT Steps alone (*n* = 28), BT Steps with non-therapist coaching (*n* = 28), and BT Steps with CBT therapist coaching (*n* = 31). Subjects were randomized using a computer-generated randomization schedule. Subjects worked on the BT Steps program at their own pace. Subjects assigned to BT Steps without coaching received a welcome and orientation call from the project manager. Subjects randomized to either of the coaching arms received the welcome call plus regularly scheduled weekly phone calls for coaching, encouragement, and support. Calls focused on the user’s progress in BT Steps, troubleshooting problems they were having with the program, and setting progress goals for the next coaching session. No formal therapy was provided by the coaches; thus, both lay and CBT coaches completed the same tasks. Two CBT coaches (DMJ and HLM) and one lay coach (RG) were used in the study. The lay coach was supervised by the CBT therapist and could consult with them as needed.

### Overview of BT Steps content

*Step 1* (‘Learning About BT Steps’) provides an overview of the program and explains the principles of treatment (exposure and response prevention (ERP)). In *Step 2* (Identifying Major Rituals and Their Costs), ERP is explained in more detail. Subjects identify their main rituals and obsessions and how much these affect their lives in terms of time and money. In *Step 3* (Identifying Triggers and Setting Goals), subjects review a list of 162 stimulus triggers to identify those that trigger their own rituals and obsessions and rate the discomfort each trigger causes. *Step 4* (Co-therapy with a Relative or Friend) helps subjects decide whether to involve a relative or friend as an exposure co-therapist and educates the co-therapist on how to best facilitate treatment. In *Step 5* (First Exposure and Ritual Prevention [E & RP]), subjects choose their first trigger and develop a detailed goal for first exposure. Coping tactics are reviewed to enable them to complete exposure and ritual prevention successfully. *Step 6* (Fine Tuning) reviews results from their exposure session and advises them how to improve subsequent sessions. Subjects can return to Step 6 as needed. Patients document results in an online diary enabling patients to track their progress and reinforce their efforts. *Step 7* (Continuing Treatment) helps subjects habituate to the next trigger on their list by performing self-exposure sessions. This step may be repeated as many times as necessary, with new triggers being added as subjects habituate to earlier ones and they learn more about planning and doing exposure and ritual prevention in a timely and efficient manner. *Step 8* (Troubleshooting) identifies problems in exposure sessions, gives tips to overcome those difficulties, and advises them how to reduce obsessive thoughts (ruminations). It can be accessed repeatedly after Step 7 has been completed at least once. *Step 9* (Firming Up Your Gains) helps subjects build on their improvements, reduces relapse risk by teaching them how to anticipate and deal with setbacks, and encourages them to participate in work/social/leisure activities in the time freed by stopping rituals and obsessions. *Each step* consists of an introductory video, written text, and various interactive exercises, depending on the purpose of the step. The *website* also includes simple navigational controls and tools that facilitate self-management of OCD symptoms. *Tools* include the Trigger Chooser, to identify personal triggers of obsessions and rituals; the Trigger Log, a tool that tracks the user’s triggers; and the Exposure Diary, which follows progress through BT Steps. Inclusion of videos (a possible total of 156 dependent on their personalized path through BT Steps), availability via the web rather than by interactive voice response (IVR) telephone plus booklet, and use of specific web tools were major changes from the original IVR-based BT Steps [48].

### Assessments

The BT Steps program administered assessments online at day 1 and then on a regular schedule every 2 weeks thereafter. The assessment battery includes the computer-administered YBOCS [68,72] (the primary outcome), the Work and Social Adjustment Scale [73], and the Depression Scale [74]. Results of these assessments were available to the subject, to permit progress tracking throughout treatment.

In addition, subjects completed two measures of user satisfaction: 1) the System Usability Scale (SUS), a reliable, well-validated 10-item scale which evaluates how ‘user friendly’ the program was from a technical perspective [75,76] and 2) a 15-item User Satisfaction Scale evaluating the *clinical content* of online programs on a 4-point scale (strongly agree, agree, disagree, strongly disagree).

Sample size was determined using .80 power to detect a 4-point difference in YBOCS change score, SD of 6.6, a .05 significance level, and planned comparisons using a one tailed *t*-test.

## Results

### Efficacy

All three treatment arms showed a significant improvement in YBOCS score from baseline to endpoint, with mean changes of 6.5, 7.1, and 6.5 for the no coaching, lay coaching, and therapist coaching arms, respectively (Table 2). These represent effect sizes (Cohens *d*) [77,78] of 1.17 (95% CI 8.62, 4.38), 1.42 (95% CI 9.37, 4.84), and 1.13 (95% CI 8.63, 4.34), respectively (all defined as ‘large’ according to Cohen) [78]. No significant differences in YBOCS change scores between the three treatment arms was observed, *F*(2) = 0.10, *p* = .904. As treatment compliance is a critical factor in treatment outcome, we examined the number of exposure exercises completed by each treatment group. No significant differences were found in the mean number of exposures completed (11.18, 10.1, and 10.8 for no coaching, lay coaching, and therapist coaching groups, respectively, (*F*(2) = 0.033, *p* = .967)). Similarly, the number of patients in each treatment arm who completed at least one exposure exercise was non-significant (24, 24, and 25, respectively (*χ*^2^(2) = 0.380, *p* = .827)). As a whole, those subjects who did at least one exposure practice (*n* = 73) did significantly better than those who did not complete any exposure sessions (*n* = 14) (mean (SD) YBOCS change of 7.5 (5.8) vs. 2.5 (4.7), respectively, *t*(86) = 3.025, *p* = .003).

**Table 2:** **Mean Yale-Brown Obsessive-Compulsive Scale (YBOCS) baseline, endpoint, and change scores by treatment group**

**Treatment arm**	**Baseline mean (SD)**	**Endpoint mean (SD)**	**Change mean (SD)**	**95% confidence interval, change**	***t***	***p***
No coaching (*n* = 28)	22.82 (3.68)	16.32 (6.97)	6.50 (5.72)	4.29, 8.72	6.014	.000
Lay coaching (*n* = 28)	22.71 (3.97)	15.61 (5.88)	7.1071 (6.12)	4.73, 9.48	6.147	.000
Therapist coaching (*n* = 31)	21.81 (4.05)	15.32 (7.04)	6.4839 (6.09)	4.25, 8.72	5.930	.000
Total (*N* = 87)						

*t* and *p* are for within subject values.

### User satisfaction

#### System usability scale

The usability of the technical features of the program (i.e., ‘user friendliness’) was evaluated using the SUS. The mean total score on the SUS was 83.5 (SD = 14.9) (scale range is 0–100 with higher score indicating greater usability). This corresponds to a score between ‘Good’ and ‘Excellent’ on the SUS (see Table 3).

**Table 3:** **Results from the user satisfaction questionnaire**

**Question**	**Strongly disagree (1) (%)**	**Disagree (2) (%)**	**Agree (3) (%)**	**Strongly agree (4) (%)**	**Mean rating**
1. The objectives of BT Steps were clear	0	0	24	76	3.8
2. BT Steps was well organized	0	0	35	65	3.7
3. BT Steps improved my understanding of my OCD	2	7	28	64	3.5
4. The material in BT Steps was presented in an interesting manner	0	10	41	48	3.4
5. There were sufficient examples and illustrations in BT Steps	0	9	38	53	3.5
6. The concepts were clearly presented and easy to understand	0	2	28	71	3.7
7. The video examples were helpful in illustrating the concepts	0	3	42	55	3.5
8. The videos with Dr. Greist were helpful in motivating me	0	5	41	54	3.5
9. The videos featuring OCD sufferers were helpful in guiding me in exposures	0	12	29	59	3.5
10. I feel capable of applying these techniques I learned in BT Steps	0	7	40	53	3.5
11. The length of BT Steps was appropriate	0	10	50	40	3.3
12. BT Steps was as effective as traditional face to face therapy in helping me with my OCD	3	19	42	36	3.1
13. I would recommend BT Steps to others	0	0	29	71	3.7
14. I enjoyed using BT Steps	0	3	44	53	3.5
15. Overall, I was satisfied with BT Steps	0	2	34	64	3.6
Mean item rating					3.5

#### User satisfaction scale

Ratings evaluating patient satisfaction with the clinical content of BT Steps are shown in Table 3. Subjects found BT Steps helpful, well organized, and presented in an interesting manner. Ninety-eight percent agreed or strongly agreed with the statement that they were satisfied with BT Steps, and all said they would recommend it to others. Interestingly, when asked which method of therapy (computer vs. clinician) they preferred, 48% said computer, 33% traditional face-to-face therapy, and 19% had no preference, *χ*^2^(2) = 7.48, *p* = .024.

#### User satisfaction with coaching

Since coaching was a unique variable in the study, we were interested in examining user satisfaction with the coaching component and whether there were any differences in satisfaction between lay coaches and CBT therapist coaches. Descriptive statistics are presented in Table 4. Overall, there was a high level of satisfaction with coaching from both groups, with no significant difference on the mean coaching satisfaction rating between groups (*p* = .216). We also were interested in whether participants in the coaching conditions felt that coaching was an essential factor in their success. Seventy-three percent ‘agreed’ or ‘strongly agreed’ with the statement that they needed coaching to succeed in the program.

**Table 4:** **Ratings of coaching satisfaction by treatment group**

**Question**	**Lay coach mean (SD)**	**Therapist coach mean (SD)**	***t***	***p***
1. Setting goals with my coach helped me stay motivated to work on BT Steps	3.6 (0.58)	3.5 (0.71)	0.66	.511
2. My coach adequately explained OCD concepts when needed	3.8 (0.38)	3.8 (0.67)	0.82	.935
3. My coach was available to answer questions outside of coaching calls	4.0 (0.36)	3.8 (0.76)	1.10	.275
4. My coach provided technical support when needed	4.0 (0.55)	3.6 (0.98)	1.51	.139
Mean item rating	3.8 (0.33)	3.7 (0.54)	1.25	.216

Scale range: 1 = strongly disagree, 2 = disagree, 3 = agree, 4 = strongly agree.

## Discussion

In the current study, the addition of coaching did not significantly improve outcomes, using either a lay coach or a CBT therapist coach. This is divergent from other studies that have found coaching beneficial, or even necessary, for positive treatment outcomes [53,58,59]. One possible reason is that while the BT Steps only group did not receive any coaching sessions, they did have an initial orientation call with the project manager. Recent studies have shown that even minimal human contact may be enough to encourage treatment compliance. In his review of the literature (‘What Makes Internet Therapy Work’) [79], Andersson found that the most critical component seemed to be that it includes some form of minimal therapist support, be that email, phone call, or live sessions. Our data seem to bear that out. The pretreatment orientation session may have made it clear to the patient that there was a person behind the support [79]. However a no-treatment control group would be necessary to confirm this hypothesis.

Another possible reason for the absence of benefit of coaching found in several earlier studies of CCBT programs was the difference in program media and components. There was a 20% greater reduction in YBOCS severity with this web version of BT Steps employing video components including exposure and response prevention vignettes compared with the original IVR version. While readily accessible via telephone, the IVR version also required correlated reading text from booklets. All text in the web version was embedded in the web program at appropriate spots, and the addition of videos and simple navigational tools was an additional difference from the original BT Steps.

Strengths of this study were inclusion of most subjects, excluding only those with significant comorbid depression, suicidal ideation, or severe OCD. The web is steadily more widely available as demonstrated by subject participation from 26 states and two ex-US countries (Canada and Singapore).

Limitations of the study include the sample being mainly college educated (66% with at least a 2-year degree, compared to a rate of 28.8% for all US citizens over 25 years old [80]). While no significant difference was found in our sample between those with and without a college degree (mean YBOCS change of 6.6 vs. 6.5, respectively), only four subjects in our sample had only a high school diploma with no college experience at all. It is unknown if similar results would be found with this cohort. It is also unknown whether similar results would be found in patients with more severe OCD, patients with significant comorbid depression, or patients with limited insight or motivation.

## Conclusions

Results support the use of online self-help for the treatment of moderate to moderately severe OCD. The addition of coaching by either a lay coach or a CBT therapist coach did not significantly improve outcomes. The implications of this finding are important: by reducing the need for live follow-up sessions, treatment becomes both more affordable and more accessible. Reduced demands on therapist’s time result in wider dissemination of treatment, since therapists can see more patients, or may see only patients not responsive to unwilling to use CCBT. Under a ‘stepped care’ model of treatment, matching the appropriate level of intervention, starting with the least restrictive and most effective, enhances treatment outcomes, controls healthcare costs, and helps allocate scare mental health resources more effectively [61-64,81]. Recent studies of stepped care in OCD found equivalent treatment outcomes to standard clinical CBT but significantly lower treatment costs [65-67]. Internet-based CBT fits nicely in the stepped care model, as patients who do not respond to email messages could be stepped up to lay coach follow-up, and from there to traditional CBT therapy, if such therapists are available.
